# The transcriptional co-repressor TLE3 suppresses basal signaling on a subset of estrogen receptor α target genes

**DOI:** 10.1093/nar/gku791

**Published:** 2014-09-15

**Authors:** Maïka Jangal, Jean-Philippe Couture, Stéphanie Bianco, Luca Magnani, Hisham Mohammed, Nicolas Gévry

**Affiliations:** 1 Département de biologie, Faculté des sciences, Université de Sherbrooke, 2500 boulevard de l'Université, Sherbrooke, Québec J1K 2R1, Canada; 2 Department of Surgery and Cancer, Imperial Centre for Translational and Experimental Medecine, Imperial College Hammersmith, London W12 0NN, UK; 3 Cancer Research UK Cambridge Research Institute, Li Ka Shing Centre, Robinson way, Cambridge CB2 0RE, UK

## Abstract

Chromatin constitutes a repressive barrier to the process of ligand-dependent transcriptional activity of nuclear receptors. Nucleosomes prevent the binding of estrogen receptor α (ERα) in absence of ligand and thus represent an important level of transcriptional regulation. Here, we show that in breast cancer MCF-7 cells, TLE3, a co-repressor of the Groucho/Grg/TLE family, interacts with FoxA1 and is detected at regulatory elements of ERα target genes in absence of estrogen. As a result, the chromatin is maintained in a basal state of acetylation, thus preventing ligand-independent activation of transcription. In absence of TLE3, the basal expression of ERα target genes induced by E2 is increased. At the *TFF1* gene, the recruitment of TLE3 to the chromatin is FoxA1-dependent and prevents ERα and RNA polymerase II recruitment to *TFF1* gene regulatory elements. Moreover, the interaction of TLE3 with HDAC2 results in the maintenance of acetylation at a basal level. We also provide evidence that TLE3 is recruited at several other regulatory elements of ERα target genes and is probably an important co-regulator of the E2 signaling pathway. In sum, our results describe a mechanism by which TLE3 affects ligand dependency in ERα-regulated gene expression via its binding restricting function and its role in gene regulation by histone acetylation.

## INTRODUCTION

Estrogen is the ligand for the nuclear receptor estrogen receptor alpha (ERα) and is implicated in various pathologies such as osteoporosis and breast, ovarian and endometrial cancers (1). Upon its induction by estrogen, ERα binds to DNA regulatory elements and activates or represses its target genes expression (2). To prevent inappropriate transcription events, ERα activity is tightly regulated by several mechanisms among which chromatin and co-factors binding play crucial roles.

Chromatin has a general repressive effect on basal transcription, but it can also play more specific roles in transcriptional regulation (3). For example, post-translational modifications of histone tails, such as acetylation, methylation, phosphorylation, sumoylation and ubiquitination, are epigenetic marks capable of deeply altering chromatin structure and influencing transcription (4,5). In 2007, a study of the epigenetic landmarks found around ERα target genes in MCF-7 cells highlighted the importance of histones post-translational modifications in ligand-dependent gene activation, particularly histone methylation. The authors showed that an H3K9me3 and H3K4me1/2 demethylase named LSD1 was present in the vicinity of most ERα-activated genes to counteract the effects of several histone methyl transferases (HMTs) whose function is to establish a chromatin state unfavorable for transcription activation. In absence of estrogen, HMTs were maintained around regulatory elements to methylate histone tails and prevent constitutive gene activation. Upon estrogen stimulation, LSD1 was recruited to remove the inhibitory marks and allow liganded ERα to stimulate the transcription of its target genes, thus demonstrating the importance of post-translational modifications of histones, particularly methylation, in ligand-dependent gene activation (6).

Genome-wide chromatin immunoprecipitation (ChIP)-seq experiments revealed that more than 50% of ERα-binding sites are localized in the vicinity of a Forkhead site (FKH) recruiting the transcription factor FoxA1 (Forkhead box protein A1) (7,8). FoxA1 is a member of the FKH family of winged helix transcription factors and is involved in the development and differentiation of several organs including liver, kidney, pancreas, lung, prostate and mammary gland (9). In breast cancer cells, FoxA1 plays an important role in the estrogen signaling pathway (7). It has been proposed that the binding of FoxA1 to chromatin promotes nucleosome remodeling (10), which allows ERα interaction with the surrounding binding sites. Hence, ERα cannot bind chromatin in absence of FoxA1. This limitation in ERα recruitment results in a significant decrease in the expression of estrogen-induced ERα target genes, illustrating the essential role of FoxA1 in the estrogen pathway (7).

Thus, the control of ERα-mediated transcription is established at several levels. Chromatin modifications and co-factor binding have to be regulated to prevent inappropriate gene activation. It is possible that this regulation can be achieved prior to ligand stimulation, by co-repressor complexes containing histone modification enzymes that prevent premature gene expression.

Groucho/Grg/TLE is a family of such transcriptional co-repressors known to be interacting partners of histone deacetylases (HDACs) (11,12,13) and FoxA1 (14,15). The members of this family share a basic structure that includes a C-terminal tandem WD40 repeat domain responsible for protein interactions, an N-terminal glutamine-rich region that mediates oligomerization and an internal Ser/Thr/Pro-rich sequence containing the nuclear localization signal and several phosphorylation sites. However, they are deprived of a DNA-binding domain (11). As such, their recruitment to target promoters occurs through direct interactions with a broad spectrum of sequence-specific DNA-binding transcription factors (16,17,18). One example is provided by the interaction of Grg3 (TLE3 mouse homolog) with FoxA1 in the liver that initiates a remodeling of nucleosomes that represses transcription, and reduces the expression of nearby genes (15). Moreover, a study in tamoxifen-resistant breast tumors showed that the expression of TLE3 was associated with longer remission periods, demonstrating a potential role for TLE3 in breast cancer progression (22). Given that TLE3 interacts with FoxA1 and that both factors are important in cancer development, we investigated their role in the MCF-7 breast cancer cell line. We hypothesized that in absence of estrogen stimulation, TLE3 could act as a co-regulator by inhibiting transcription once tethered to DNA in the absence of estrogen stimulation.

Here, we show that TLE3 is indeed an important regulator of ERα target genes transcription. It is an essential factor that maintains transcription in a basal state in the absence of hormone. TLE3 is recruited to the chromatin by FoxA1 at several ERα binding sites throughout the genome. It mediates repression via interaction with HDACs to regulate histone acetylation, which in turn affects the recruitment of transcription factors, RNA polymerase II (RNAPII) and co-activators to regulatory regions.

## MATERIAL AND METHODS

### Cell culture and shRNA lentiviral transduction

MCF-7 cell lines were maintained in DMEM (Wisent) containing 10% fetal bovine serum (FBS) and antibiotics (penicillin and streptavidin). Prior to their use in E2 treatment experiments, MCF-7 cells were grown in DMEM without phenol red (Wisent) and supplemented with 5% charcoal-dextran-treated FBS for at least 3 days. These cells were mycoplasma free and were kept in a humidified chamber at 37°C in 5% CO2. 17β-estradiol, TAM and ICI 182780 (Sigma) were used at concentrations of 10^−7^, 10^−5^ and 10^−5^ M, respectively, unless otherwise stated.

The shRNA sequences (listed in Supplementary Table S1 for shTLE3 and (23) for shFoxA1) designed to inhibit TLE3 (Open Biosystems) and FoxA1 were cloned into the pLKO.1 and pLVTHM backbones, respectively. shRNA lentiviruses were obtained by co-transfection of pLKO.1 or pLVTHM vectors, pMD2G, and psPAX2 into the human 293T cell line. Upon harvesting the viruses, 80 μg of polybrene 1000x was added for every 10 ml of virus.

### Protein immunoprecipitation (IP)

MCF-7 cells were hormone-deprived for at least 3 days and then treated with vehicle (EtOH 0,1%) or E2 (10^−7^ M) for 30 min. After 10 min of crosslink with formaldehyde 1.1%, the cells were harvested in 200 μl of SDS lysis buffer (1% SDS, 10 mM EDTA, 50 mM Tris pH 8.1) and put on ice for 30 min. Cell lysis was followed by two rounds of sonication (amplitude of 40% for 15s, 3 min on ice between each sonication round), to decrease DNA viscosity. The volume of each sample was completed to 1 ml with IP dilution buffer (0.01% SDS, 1.1% Triton, 1.2 mM EDTA, 16.7 mM Tris ph 8.1, 167 mM NaCl) and 50 μl of each sample were spared for the input. The antibodies were added as shown in Supplementary Table S2, and incubated overnight at 4°C. 40μl of magnetic beads (Dynabeads protein A, Novex by Life Technologies) were added in each IP reaction for 4 h. Beads were washed two times with TSE 150 (0.1% SDS, 1% Triton, 2 mM EDTA, 20 mM Tris ph 8.1, 150 mM NaCl), then two times with TSE 500 (0.1% SDS, 1% Triton, 2 mM EDTA, 20 mM Tris ph 8.1, 500 mM NaCl) and finally two times with TE1x. IPs and inputs were eluted in 200 μl of elution buffer (0.1 M NaHCO_3_, 1% SDS) for 30 min at room temperature and the crosslink was reverted by incubating overnight at 65°C. SDS loading dye was added to the samples and incubated 5 min at 95°C before loading the gels used for western blot.

### ChIP assays

MCF-7 cells were hormone-deprived for at least 3 days and then treated with vehicle (EtOH 0.1%) or E2 (10^−7^M) for 30 min. ChIP assays were performed essentially as described previously (24) with a panel of specific antibodies listed in the Supplementary Table S2. qPCR was performed by comparing immunoprecipitated DNA to a standard curve obtained from total DNA. The primers used are listed in the Supplementary Table S3.

### RNA analysis

Cells grown for 3 days in estrogen-free medium were treated for 3 h with vehicle (ethanol 0.1%) or estrogen (10^−7^M). Total RNA was isolated using GenEluteTM Mammalian Total RNA Miniprep Kit (Sigma Aldrich) and the RNeasy kit (Qiagen). RNA was reverse-transcribed into first-strand cDNA using M-MLV reverse transcriptase (Enzymatics). Samples were subjected to q-PCR using a Bio-Rad C1000TM Thermal cycler. The relative abundance of all the tested genes was calculated after normalization to *GapDH* (Glyceraldehyde 3-phosphate dehydrogenase) mRNA, where relative expression levels were calculated as 2-ΔCT where ΔCT = CT test gene – CT GapDH. Primers used are listed in the Supplementary Table S3.

### RNA-Seq and ChIP-Seq libraries

RNA-Seq libraries were prepared essentially as described previously (25). For the bioinformatics analysis, we merged the data obtained from two experiments. ERα ChIP-Seq libraries were prepared essentially as described previously (26).

### Bioinformatics analysis

*In silico* studies: Expression correlation analysis between ERα and TLE3 in breast cancer cells was achieved using Oncomine (https://www.oncomine.com) and GOBO (27).

RNA Seq: Fastq files were aligned against the reference human genome hg18 using BWA (28). Only sequence reads that were uniquely mapped to the genome with a mapping quality score >10 were used. Two biological replicates were performed and merged for subsequent analyses. The estimation of transcript abundance was determined using Cufflinks (29). The overlap of genes affected by E2 and shTLE3 was determined using BioVenn (30), and listed in Supplementary Table S4–S8. Gene ontology analyses were performed using GeneCoDis3 (31), and listed in Supplementary Table S9.

ChIP-Seq: From the MCF-7 cell line, we collected raw datasets for FoxA1, HDAC2 and TLE3 in complete medium ((32,33,34) respectively). All the ChIPSeq data were generated as follows: generated reads were aligned against the human reference genome hg18 using BWA. Only sequence reads that were uniquely mapped to the genome with a mapping quality score >10 were used. Two biological replicates were performed and merged for subsequent analyses. Peaks were called by MACS using default parameters. Binding sites overlaps were determined using BioVenn. Aligned tags were converted to WIG files then bigWIG files by F-Seq (35) and visualized with IGV (36).

## RESULTS

### TLE3 is involved in the regulation of ERα target gene transcription

To explore the link between ERα status and TLE3 expression in breast tumors, we performed an *in silico* analysis from publicly available data. The resulting expression profile shows that TLE3 and ERα are co-expressed. Indeed, the expression of TLE3 was stronger in ERα-positive breast cancer cell lines compared to ERα-negative cell lines (Supplementary Figures S1A and S1B). This co-expression was also observed in breast cancer primary tumors (Supplementary Figure S1C). These observations suggest that TLE3 and ERα expression are associated despite the fact that the TLE3 gene is not regulated by estrogen (E2) (Supplementary Figure S2A and S2B).

**Figure 1.**
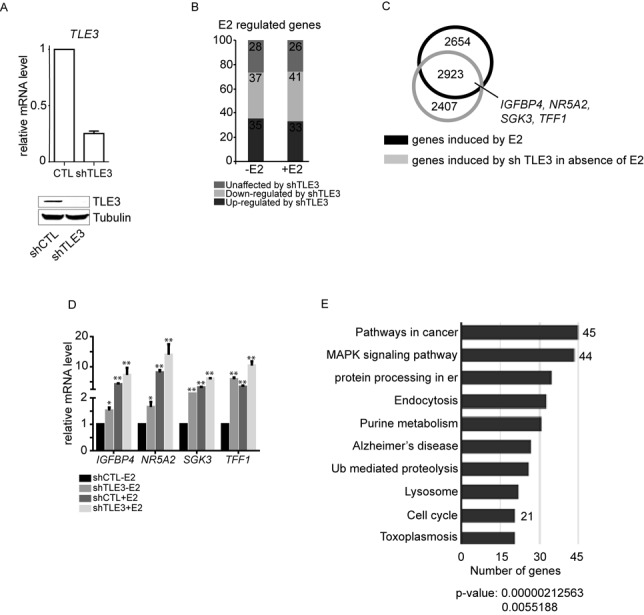
TLE3 is involved in the regulation of ERα target genes transcription. (**A**) RT-qPCR and western blot analysis of MCF-7 cells infected with lentiviruses expressing no (shCTL) or one shRNA (shTLE3) directed against TLE3. TLE3 mRNA expression was normalized to *GapDH* and Tubulin was used as the leading control for the western blot. (**B**) Histogram representing the distribution of genes affected by the knockdown of TLE3. The genes regulated by E2 were compared to the genes up- and down-regulated by shTLE3 in absence or in presence of E2. (**C**) Venn diagram of genes up-regulated by E2 and genes up-regulated by the knockdown of TLE3 in absence of E2. (**D**) MCF-7 cells were infected with an empty lentivirus (shCTL) or a lentivirus carrying an shRNA against human TLE3 (shTLE3) and treated with vehicle (-E2, ethanol 0.1%) or 17β-estradiol (+E2 10^−7^M) for 3 h. Total RNA was used to generate cDNA for RT-qPCR. The results presented are average of at least three independent experiments (Student's *t*-test * = *P* < 0.05; ** = *P* < 0.01) (E) Gene ontology of the overlapping genes reported in the Venn diagram in (C). er: endoplasmic reticulum; Ub: ubiquitin.

TLE3 and ERα expression levels are correlated and these factors are also interaction partners ((34) and Supplementary Figure S3A and S3C). Thus, we supposed that TLE3 might be an important regulator of ERα target gene expression. To test this hypothesis, we knocked down TLE3 in MCF-7 cells using a short hairpin-RNA (shRNA) (Figure 1A) and proceeded with mRNA deep sequencing (RNA-Seq). The bioinformatics analysis revealed that the majority of E2-regulated genes are affected by the depletion of TLE3. More precisely, in absence of E2, 35% (3141) were up-regulated, 37% (3475) were down-regulated and 28% (2299) were not affected by the knockdown of TLE3. After an E2 treatment, 33% (3569) of the ERα target genes were up-regulated, 41% (4457) were down-regulated and 26% (2792) were unaffected by the depletion of TLE3 (Figure 1B). Upon TLE3 depletion, the expression of 35% of ERα target genes is enhanced supporting the idea that TLE3 could be a co-repressor of ERα activity. However, the knockdown of TLE3 also decreased 41% of ERα target genes expression suggesting a co-activator role for TLE3 in ERα transcription pathway. These results are in agreement with a previous study that reported a bivalent role for TLE3 during transcription (37). In our study, we focused our attention on the co-repressor role of TLE3.

To examine the importance of TLE3 in the inhibition of ERα target genes transcription in the absence of hormone, we generated a Venn diagram with the genes up-regulated by E2 and the genes up-regulated by TLE3 knockdown in absence of E2 (Figure 1C). This revealed that 52.4% of genes whose expression was enhanced by E2 were also induced in TLE3-depleted cells in absence of hormonal treatment whereas 16.1 and 31.5% of genes induced by E2 are repressed or unaffected by TLE3 knockdown, respectively (Supplementary Figure S4A and S4B). We validated these observations on a subset of these genes using RT-qPCR on several genes whose expression is affected by the depletion of TLE3 (Figure 1D, Supplementary Figures S5 and S6). In absence of TLE3, the RT-qPCR experiments showed a 2-fold increase in basal mRNA levels for *IGFBP4*, *NR5A2* and *SGK3* compared to the control, whereas *TFF1* mRNA levels increased 7-fold under the same conditions. Upon induction with E2, the expression of these genes increased in both control and TLE3-depleted cells (Figure 1D). These results show that the basal expression of about half of ERα target genes, usually induced by E2, is enhanced in absence of TLE3, strengthening the idea that TLE3 could be a co-regulator of ERα activity in breast cancer cells.

Finally, in order to explore the biological significance of the genes whose expression is induced both by E2 and TLE3 depletion, we performed a gene ontology analysis. Among others, a significant enrichment was observed in three mechanisms undoubtedly crucial for cancer development and proliferation: pathways in cancer, MAPK signaling pathways and cell cycle (Figure 1E). Taken together, these results support the importance of TLE3 in the regulation of E2 target genes transcription, and its putative role in breast cancer biology.

### TLE3 is recruited to the regulatory elements of ERα target gene

**Figure 2.**
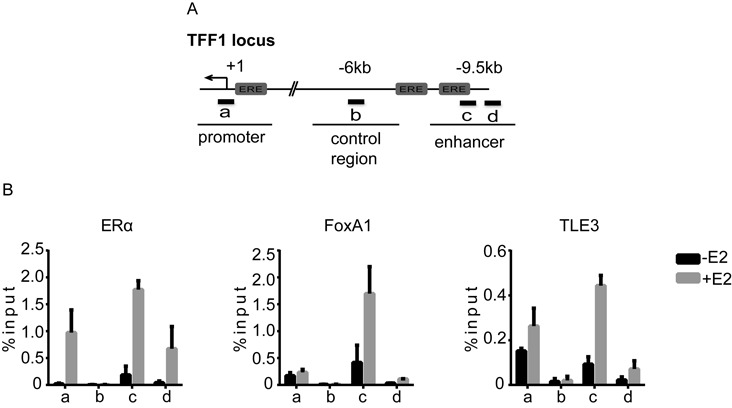
TLE3 is recruited to *TFF1* regulatory elements. (**A**) Map of the *TFF1* gene showing its regulatory elements and the qPCR primers (identified by the letters a, b, c, and d) used for chromatin immunoprecipitation (ChIP) analysis. (**B**) MCF-7 cells were treated with vehicle (-E2, ethanol 0,1%) or 17β-estradiol (+E2, 10^−7^ M) for 30 min. Cells were crosslinked and subjected to ChIP with antibodies raised against ERα, FoxA1 and TLE3. Isolated DNA was analyzed with the primers described in (A). The data shown are the mean ± SEM of at least three independent experiments*.*

The estrogen-responsive *TFF1* promoter has been extensively studied as a model system to understand the regulation of transcription by ERα. Moreover, we showed that *TFF1* is one of the genes highly induced by the knockdown of TLE3 in absence of hormonal treatment (Figure 1D). To better understand the mechanism behind the role of TLE3 in the regulation of ERα target gene transcription, we examined the recruitment of TLE3 to the *TFF1* gene. MCF-7 cells were treated with E2 for 30 min, and then subjected to ChIP followed by qPCR. Figure 2A provides a graphic representation of *TFF1* regulatory elements and the position of the primers used for the qPCR. We designed four pairs of primers (identified in Figure 2A by thick black lines): primer ‘a’ located directly on the proximal promoter (arrow); primer ‘b’ in the control region where none of the studied transcription factors are expected to bind (as indicated by our results and published ChIP-sequencing (ChIP-Seq) experiments, Supplementary Figure S7); primer ‘c’ located in the enhancer; primer ‘d’ bordering the enhancer. In absence of E2, we found that FoxA1 and TLE3 were bound to the enhancer and promoter of *TFF1*. E2 stimulation resulted in ERα binding at *TFF1* regulatory regions, and the enhancement of FoxA1 and TLE3 recruitment. As expected, no recruitment of these factors to the control region was observed (Figure 2B). These results suggest that since TLE3 is recruited to *TFF1* regulatory elements along with ERα and FoxA1, this factor is likely directly involved in the regulation of *TFF1* expression.

**Figure 3.**
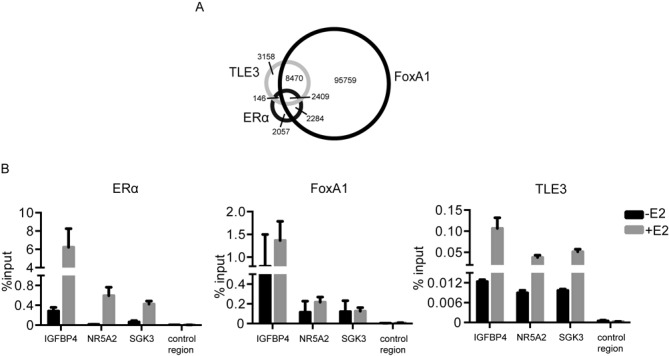
FoxA1, ERα and TLE3 share several binding sites throughout the genome. (**A**) Venn diagram representing the shared binding sites of FoxA1, ERα and TLE3. The ChIP assays were performed in MCF-7 cells grown in full medium. (**B**) ChIP assays in MCF-7 cells after treatment with vehicle (-E2, ethanol, 0.1%) or E2 (+E2, 10^−7^M) for 30 min with antibodies raised against ERα, FoxA1 and TLE3 at the regulatory elements of *IGFBP4*, *NR5A2* and *SGK3* (primers described in Supplementary Figure S4) and the control region of *TFF1* locus (primer b). The results presented are average of at least two independent experiments (Student's *t*-test * = *P* < 0.05; ** = *P* < 0.01).

FoxA1 is essential for the regulation of ERα target genes transcription and shares about half of ERα-binding sites (7). To explore the genome-wide link between ERα, FoxA1 and TLE3 binding, we compared ERα, FoxA1 (32) and TLE3 (34) ChIP-Seq datasets from MCF-7 cells. The Venn diagram in Figure 3A illustrates the overlap between FoxA1-, ERα- and TLE3-binding sites. As expected, over half of ERα-binding sites were shared by FoxA1 (68%). Among the shared sites, 51% also overlapped with TLE3, supporting the idea that TLE3 might be recruited to other ERα target genes than *TFF1*. To validate these observations, we designed primers (Supplementary Figure S8) that are located in the nearest ERα-binding sites around *IGFBP4*, *NR5A2* and *SGK3*, three genes whose expression is induced by the depletion of TLE3 in absence of hormone, (Figure 1D). We evaluated the recruitment of ERα, FoxA1 and TLE3 by ChIP and found that ERα, FoxA1 and TLE3 are already present at these sites in absence of E2, and that their recruitment is enhanced in presence of E2 (Figure 3B). Taken together, these results show that the co-localization of ERα, FoxA1 and TLE3 is observed in several regulatory regions of other ERα target genes, and suggest that some of the genes regulated by ERα and FoxA1 could also be regulated by TLE3.

### FoxA1 is necessary for TLE3 recruitment on chromatin

**Figure 4.**
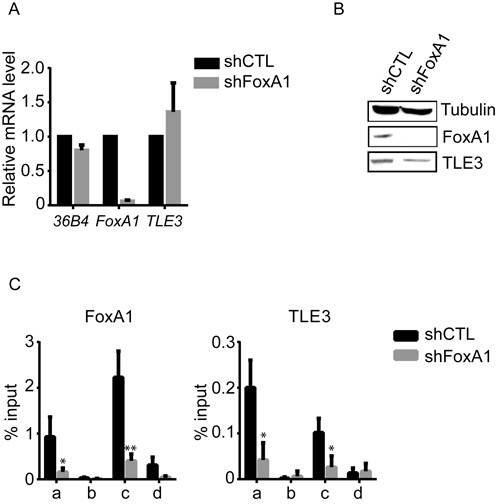
FoxA1 is essential for TLE3 recruitment at *TFF1* regulatory elements. RT-qPCR (**A**) and western blot (**B**) analysis of MCF-7 cells infected with an empty lentivirus (ShCTL) or a lentivirus carrying an shRNA against FoxA1 (shFoxA1). RNA levels were normalized to 36B4 and Tubulin was used as a loading control. (**C**) FoxA1 and TLE3 occupancy measured by ChIP at *TFF1* regulatory elements, as described in Figure 2A, in MCF-7 cells depleted (shFoxA1), or not (shCTL), in FoxA1 in absence of E2 stimulation. The data shown are the mean ± SEM of at least three independent experiments (Student's *t*-test * = *P* < 0.05; ** = *P* < 0.01)*.*

TLE3 seems to be directly involved in the regulation of ERα target genes transcription (Figure 1D). However, since TLE3 has no DNA-binding domain, it needs to be recruited to DNA via other factors (16,17,18). Two studies have previously shown that FoxA1 might be involved in leading TLE3 to DNA (14,15). Moreover, the interaction between TLE3 and FoxA1 was demonstrated in mouse liver cells (15) and in our breast cancer cells (Supplementary Figures S3B and S3C). Thus, we hypothesized that FoxA1 might also be responsible for TLE3 recruitment to chromatin in MCF-7 cells. To test this, we knocked down FoxA1 using targeted shRNA, and analyzed TLE3 binding to chromatin by ChIP. FoxA1 depletion did not affect *TLE3* mRNA expression (Figure 4A), nor protein level (Figure 4B), but considerably reduced FoxA1 and TLE3 binding to *TFF1* regulatory elements (Figure 4C), arguing that FoxA1 is essential for TLE3 recruitment to chromatin in MCF-7 cells.

### The depletion of TLE3 promotes a transcriptionally active chromatin

**Figure 5.**
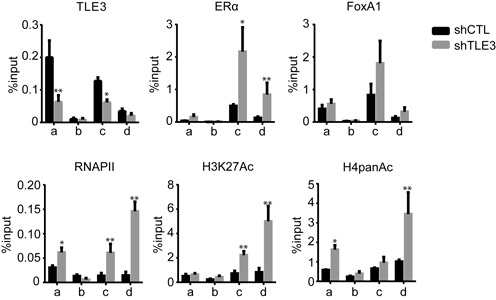
TLE3 hinders the recruitment of ERα, FoxA1 and RNAPII and reduces the acetylation of histones at *TFF1* regulatory elements in absence of estrogen. TLE3, ERα, FoxA1 and RNAPII occupancy and H3K27Ac and H4PanAc relative abundance at *TFF1* regulatory elements, as described in Figure 2A, measured by ChIP in MCF-7 cells grown in estrogen-free medium and depleted (shTLE3) or not (shCTL) in TLE3 The results presented are average of at least three independent experiments (Student's *t*-test * = *P* < 0.05; ** = *P* < 0.01).

To determine the effects of TLE3 knockdown on the conformation of chromatin at the enhancer in a basal state, we first assessed the recruitment of ERα, FoxA1 and RNAPII to *TFF1* regulatory elements. To this aim, a ChIP analysis was performed on MCF-7 cells depleted in TLE3. As expected, TLE3 recruitment was reduced when compared to the control. The recruitment of ERα, FoxA1 and RNAPII to *TFF1* enhancer was slightly increased in absence of TLE3 in untreated cells, consistent with the enhancement of gene expression observed under the same conditions (Figure 1D). This augmentation was also observed at the *TFF1* promoter but to a lesser extent (Figure 5). However, this increase in ERα and FoxA1 recruitment is not as important on the other ERα target genes as for *TFF1* (data not shown).

To identify the mechanism by which TLE3 might act as a co-repressor, we also evaluated the changes in two post-translational modifications that mark active chromatin elements: acetylation (Ac) of histone H3 lysine 27 (H3K27), and the general level of histone H4 acetylation (H4panAc). As a control of histone loading, we performed a ChIP assay of H3 (Supplementary Figure S9). ChIP in TLE3-depleted cells cultured in E2-stripped medium revealed that acetylation of H3K27 and H4pan was enhanced at the distal estrogen response element (ERE) of the *TFF1* locus (primer c), but not at the control region when compared to control cells (Figure 5). These results suggest that TLE3 is indeed implicated in the regulation of histone acetylation at ERE.

### TLE3 regulates histone acetylation by recruiting HDAC to the chromatin

**Figure 6.**
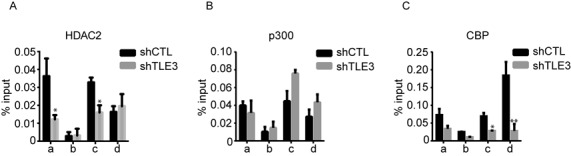
TLE3 regulates histone acetylation by recruiting HDAC to chromatin. ChIP assays at *TFF1* regulatory elements, as described in Figure 2A, for HDAC2, p300 and CBP in MCF-7 cells grown in estrogen-deprived medium in absence (shTLE3) or in presence (shCTL) of TLE3. The data shown are the mean ± SEM of at least three independent experiments (Student's *t*-test * = *P* < 0.05; ** = *P* < 0.01).

The increase in histone acetylation observed in absence of TLE3 could be due to a decrease in HDAC recruitment, an increase of histone acetyl transferase (HAT) activity, or both. The interaction of TLE3 with HDAC has already been shown (12) and our IP experiments, strengthen the idea that TLE3, FoxA1 and HDAC2 form a complex in absence of estrogen in MCF-7 cells (Supplementary Figures S3B and S3C). Thus, to verify whether the recruitment of HDAC in MCF-7 cells is indeed TLE3-dependent, we analysed ChIP-Seq data of HDAC2 in MCF7 cells (33) and verified its presence on *TFF1* regulatory elements (Supplementary Figure S10). We then validated these results using ChIP experiments for HDAC2 at *TFF1* regulatory elements, in TLE3-depleted MCF-7 cells grown in hormone-deprived medium. In absence of TLE3, HDAC2 recruitment to chromatin was decreased, suggesting that TLE3 regulates acetylation by its interaction with HDAC (Figure 6A). To evaluate HAT activity, we examined by ChIP the recruitment of p300 and CBP to *TFF1* regulatory elements under the same conditions. p300 and CBP are HAT that bind cell-specific enhancers (38) and are responsible for H3K27 acetylation (39,40,41). In TLE3-depleted cells, the recruitment of these HAT did not increase compared to control, suggesting that p300 and CBP are not the primary factors responsible for the increase of histone acetylation in absence of TLE3 (Figure 6B and 6C).

Together, these results indicate that TLE3 is a co-regulator of ERα activity in absence of E2. The restriction of ERα and RNAPII binding to the chromatin and also histone acetylation prevention by favoring HDAC recruitment appear to be two mechanisms involved in this function.

## DISCUSSION

Several studies have examined the role of TLE3 and homolog factors in many organisms including yeast (Tup1), *Drosophila* (Groucho), mouse (Grg3) and human (TLE3). In human, TLE3 function has been explored in various cell lines, namely, adipocytes (37), prostate cancer cells (21), ovarian carcinoma cells (42) and breast cancer cells (22). In breast cancer, the progression-free survival of patients with ERα+ tumors is positively correlated with TLE3 expression (22). However, little information was collected on the molecular role of this factor and how it interferes with ERα activity. Here, we show that at the *TFF1* locus, FoxA1 binds to chromatin and interacts with TLE3 when it is present. The interaction of TLE3 with HDAC prevents the inappropriate acetylation of histones around the regulatory regions, as well as ERα and RNAPII recruitment. Under these conditions, ERα target genes cannot be transcribed without hormonal stimulation. However, in absence of TLE3, FoxA1 remodels the chromatin into an active state, allowing ERα and RNAPII tethering and subsequent expression of ERα target genes without any hormonal control. Thus, in absence of TLE3, cells could be more prone to inappropriate gene expression, leading to uncontrolled proliferation and, possibly, to cancer development. Our study at the *TFF1* locus, leads us to propose that the mechanism could be the same around other E2 regulated genes (Supplementary Figures S5, S6 and S8).

In absence of E2, the depletion of TLE3 causes an increase in the basal expression of a significant subset of ERα target gene basal expression. This induction could be ERα-dependent or the consequence of another pathway activated by the depletion of TLE3. To test this hypothesis, we inhibited ERα activity with fulvestrant (ICI182780) in absence of TLE3 and examined *TFF1* basal expression. *TFF1* mRNA level was lower in TLE3-depleted cells after treatment with ICI (Supplementary Figure S11), demonstrating the importance of ERα in the basal induction of its target genes in TLE3-depleted cells. In breast cancer cells, the phosphorylation of ERα is an essential mechanism for ligand-independent transcriptional activation of ERα (43). Indeed, ERα can be stimulated even in absence of E2 by a variety of growth factors. Several serine residues found in the AF-1 domain are targeted by diverse kinases, among which is the mitogen-activated protein kinase (MAPK)/extracellular signal-regulated kinase (ERK), activated by epidermal growth factor (EGF) (44). Furthermore, growth factor-stimulated breast cancer proliferation is dependent on ERα (43) and this crosstalk between ERα and growth factors is an important aspect of resistance in breast cancer therapy (45). Thus, we suggest that in absence of TLE3, ERα could be phosphorylated by kinases (possibly MAPK, Figure 1E) and induce the basal transcription of its target genes.

In several hormone therapy-resistant cancer cells, ERα is recruited to the DNA and activates its target genes in absence of E2 (45). The resistance may in part be explained by the capacity of ERα to tether to the chromatin and induce the expression of genes involved in cell growth independently of a hormonal signal. Since our results indicate that TLE3 is an important regulator of ERα basal transcription, a disturbance in its repressive function could favor tumor development and hormone-therapy resistance.

TLE3 and its homologs have commonly been described as co-repressors because they down-regulate their target genes expression. However, recent studies in adipocytes and breast cancer cells showed that theses co-factors can be co-activators as well as co-repressors. In breast cancer cells, TLE1 binds the chromatin at ERα-binding sites and is required for optimal ERα recruitment. The absence of TLE1 leads to a decline in ERα binding, thereby preventing ERα target genes expression. Thus, TLE1 appears to function as a positive regulator of ERα-mediated transcription (19). Likewise, TLE3 can also be a co-activator of transcription. In the pre-adipocyte, TLE3 is recruited at the promoter of peroxysome proliferator-activated receptor gamma (PPAR-γ) target genes and promotes their transcription. At the same time, it competes with β-catenin for transcription factor 4 (TCF4) interaction to relieve the repression by the Wnt pathway, and induces the genes responsible for adipocytes differentiation (37). Thus, in adipogenesis, TLE3 is a co-activator of PPAR-γ pathway and a co-repressor of the Wnt pathway. Our RNA-Seq analysis in MCF-7 cells supports this dual role for TLE3 as it revealed a co-activator role for TLE3 (41% of genes were down-regulated in presence of E2 in TLE3-depleted cells, Figure 1B) in addition to its co-repressor role. Moreover, after E2 treatment, the ChIP experiments showed an increase in TLE3 recruitment to the regulatory elements of *TFF1*, despite the fact that the expression of this gene is induced by the hormone. As we focused on ERα target genes repressed by TLE3, we expected a decrease of TLE3 recruitment to the enhancer and the promoter. Taken together, these results suggest that TLE3 may still be expressed in cancer cells because of its dual role in transcription.

Previous studies in *Drosophila* have shown that the repressive function of TLE3 is weakened when it is phosphorylated (46). Several phosphorylation sites situated in the central region of TLE3 can be targeted by cdc2, casein kinase 2 or MAPK activated via epidermal growth factor receptor and fibroblast growth factor receptor pathways (13). Moreover, another study showed that TLE3 interacts with HDACs in drosophila. HDACs are a family of co-repressors responsible for histone deacetylation that prevent transcription factor from accessing the chromatin thereby inhibiting transcription. An immunoprecipitation experiment, with prior phosphatase alkaline treatment of the total extract, showed that the interaction between TLE3 and HDAC is stronger when the samples were submitted to a dephosphorylation step before immunoprecipitation (data not shown). This result is consistent with the hypothesis that, in a manner similar to what is observed in drosophila, the phosphorylation of TLE3 lessens its repressive function in MCF-7 breast cancer cells. Therefore, it is possible that, in absence of E2, TLE3 is unphosphorylated and has a co-repressor activity as a result of its interaction with HDAC.

TLE3 prevents the inappropriate acetylation of histones via its interaction with HDACs and our ChIP assays demonstrated the presence of HDAC2 on *TFF1*. However, TLE3 might not interact with only one specific HDAC. Indeed, in *Drosophila*, Groucho proteins interact with Rpd3 (12), a homolog of HDAC1, 2, 3 and 8. Furthermore, an immunoprecipitation experiment conducted in our laboratory showed that TLE3 interacts with HDAC1 in MCF-7 cells (data not shown). Moreover, the analysis of TLE3 and HDAC2 ChIP-Seq data also showed that more than half of TLE3-binding sites are shared with HDAC2 (65.6%, data not shown), highlighting the importance of TLE3 interaction with HDAC2.

We also tested the recruitment of p300 and CBP to *TFF1* regulatory elements since the acetylation of their target residue, H3K27, was strongly enhanced in absence of TLE3. However, the depletion of TLE3 could also increase the acetylation of other H3 residues, or the recruitment of other HATs that could also acetylate H4 lysines. Although we focused our study on the acetylation level of H3K27, p300 is also responsible for the acetylation of H3K14, H3K18, H4K5 and H4K8. Further analysis on the acetylation of those residues should give a better picture on the role of p300. Likewise, H3K9, K14 and K18 are acetylated by GCN5 and PCAF, whereas Tip60 is responsible for H3K14 and H4K5, K8, K12 and K16 acetylation (47), suggesting that other HAT could be responsible for the elevation of the general acetylation level. Indeed, histone acetylation is governed by a complex network of several HATs interacting with numerous lysine residues, making it difficult to pinpoint the mechanism that takes place during the initiation of transcription. Thus, we speculate that TLE3 might influence the acetylation of several histones amino acid residues, and the recruitment of more than one HAT.

In conclusion, we report that TLE3 is an essential factor for the regulation of ERα target gene transcription via its function on histone acetylation. A previous study showed that HMTs and histone demethylases are responsible for the ligand-dependent transcription of ERα target genes (6). Here we demonstrated that the regulation of histone acetylation is essential for the maintenance of ERα target gene transcription in a basal state. We also showed that FoxA1, TLE3 and factors responsible for chromatin remodeling are essential determinants of ligand-dependent ERα target gene activation.

## SUPPLEMENTARY DATA

Supplementary Data are available at NAR Online.

SUPPLEMENTARY DATA
