# Supplementary material for: The transcriptional co-repressor TLE3 suppresses basal signaling on a subset of estrogen receptor α target genes
Source: Nucleic Acids Res. 2014 Sep 15;42(18):11339–48. doi: 10.1093/nar/gku791 (PMC4191390; doi:10.1093/nar/gku791)
Supplement: SUPPLEMENTARY DATA [file supp_42_18_11339__index.html]

The transcriptional co-repressor TLE3 suppresses basal signaling on a subset of estrogen receptor α target genes — The transcriptional co-repressor TLE3 suppresses basal signaling on a subset of estrogen receptor α target genes — SUPPLEMENTARY DATA 

# The transcriptional co-repressor TLE3 suppresses basal signaling on a subset of estrogen receptor α target genes

## SUPPLEMENTARY DATA

**Files in this Data Supplement:**

- SUPPLEMENTARY DATA
- SUPPLEMENTARY DATA
