# Supplementary material for: The transcriptional co-repressor TLE3 suppresses basal signaling on a subset of estrogen receptor α target genes
Source: Nucleic Acids Res. 2014 Sep 15;42(18):11339–48. doi: 10.1093/nar/gku791 (PMC4191390; doi:10.1093/nar/gku791)
Supplement: SUPPLEMENTARY DATA [file supp_gku791_nar-03588-x-2013-File008.pdf]

FIG. S1. ERα and TLE3 are co-expressed in ER+ breast cancer cells. Expression profiles from ER+ and ER- breast tumors reveals that TLE3 mRNA levels are correlated with ERα mRNA expression in ER+ breast tumors (meta-analysis conducted using Oncomine, pvalue=1.58e-29). (B) GOBO analysis (http://co.bmc.lu.se/gobo) of ERα and TLE3 mRNA level in ER+ and ER- breast cancer cells. (C) GOBO analysis of ERα and TLE3 mRNA level in various breast cancer tumors.

FIG. S2. TLE3 expression is not affected by E2. (A) Western blot and (B) RT-qPCR on total protein or mRNA extract from MCF-7 cells treated with E2 (+E2 10^{-7}M) or vehicle (-E2, EtOH 0.1%) for 3hours. TLE3 expression was normalized over GapDH. *The results presented are average of at least three independent experiments.*

FIG. S3. TLE3 interacts with ERα, FoxA1 and HDAC2. Protein IP of ERα (A), FoxA1 (B) and TLE3 (C) in MCF7 grown 3 days in estrogen free medium and treated with vehicle (EtOH 0.1%) or estrogen (10^{-7}M) for 30 minutes. The IP and western blots were realised using antibodies listed in Table S2.

FIG. S4. A subset of E2-regulated genes are down-regulated or unaffected by the knockdown of TLE3. Venn diagram of genes up-regulated by E2 and genes down-regulated (A) or unaffected (B) by the knockdown of TLE3 in absence of E2.

FIG. S5. A subset of E2 up-regulated genes recruits ERα, FoxA1 and TLE3 on their regulatory elements. RT-qPCR on total protein or mRNA extract from MCF-7 cells treated

with E2 (+E2 10^{-7}M) or vehicle (-E2, EtOH 0.1%) for 3 hours. mRNA expression was normalized over GapDH. *The results presented are average of at least three independent experiments.*

FIG. S6. A subset of ERα down-regulated genes recruits ER, FoxA1 and TLE3 on their regulatory elements. RT-qPCR on total protein or mRNA extract from MCF-7 cells treated with E2 (+E2 10^{-7}M) or vehicle (-E2, EtOH 0.1%) for 3 hours. mRNA expression was normalized over GapDH. *The data shown are the mean ± s.e.m of at least three independent experiments.*

FIG. S7. ERα, FoxA1 and TLE3 are recruited on *TFF1* regulatory elements. ChIP Seq analysis of ERα, FoxA1 and TLE3 in MCF-7 cells grown in full medium. Genomic location were obtained using Integrative Genomic Viewer (IGV 2.0). The black peaks represent the recruitment intensity of the factor. Below, the map of *TFF1* is represented with the primers used for qPCR (a, b, c and d).

FIG. S8. ERα, FoxA1 and TLE3 share other binding sites throughout the genome. ChIP Seq analysis of ERα, FoxA1 and TLE3 on the ER binding sites around IGFBP4, NR5A2 and SGK3. Genomic location were obtained using Integrative Genomic Viewer (IGV 2.0) The black peaks represent the recruitment intensity of the factor. The red line highlights the region used to design the primers.

FIG. S9. The presence of H3 in the nucleosome is not affected by the depletion of TLE3. ChIP assay on H3 at *TFF1* regulatory elements in presence (shCTL) or in absence (shTLE3)

of TLE3 in MCF7 cells grown in estrogen deprived medium. *The results presented are average of at least three independent experiments.*

FIG. S10. HDAC2 is recruited on *TFF1* regulatory elements. ChIPseq data for HDAC2 binding events around *TFF1* gene in MCF-7 cells grown in complete medium. Genomic location were obtained using Integrative Genomic Viewer (IGV 2.0). Below, a reminder of *TFF1* map with the primers used for the ChIP-qPCR experiments.

FIG. S11. ERα is responsible for the basal activation of its target genes in absence of TLE3. (A) RT-qPCR of TLE3 and *TFF1* in absence of estrogen, in MCF-7 cells depleted (shTLE3) or not (shCTL) in TLE3. The cells were grown in estrogen deprived medium for 3 days and treated with vehicle (-ICI, EtOH 0.1%) or ICI182780 (+ICI, 10^{-5}M) for 24 hours before RNA extraction. mRNA levels were normalized over GapDH. (B) Western blot analysis of ERα expression in the same conditions as in (A). Tubulin was used as a loading control. *The results presented are average of at least three independent experiments.*

Jangal et al. 2014_FigS1.

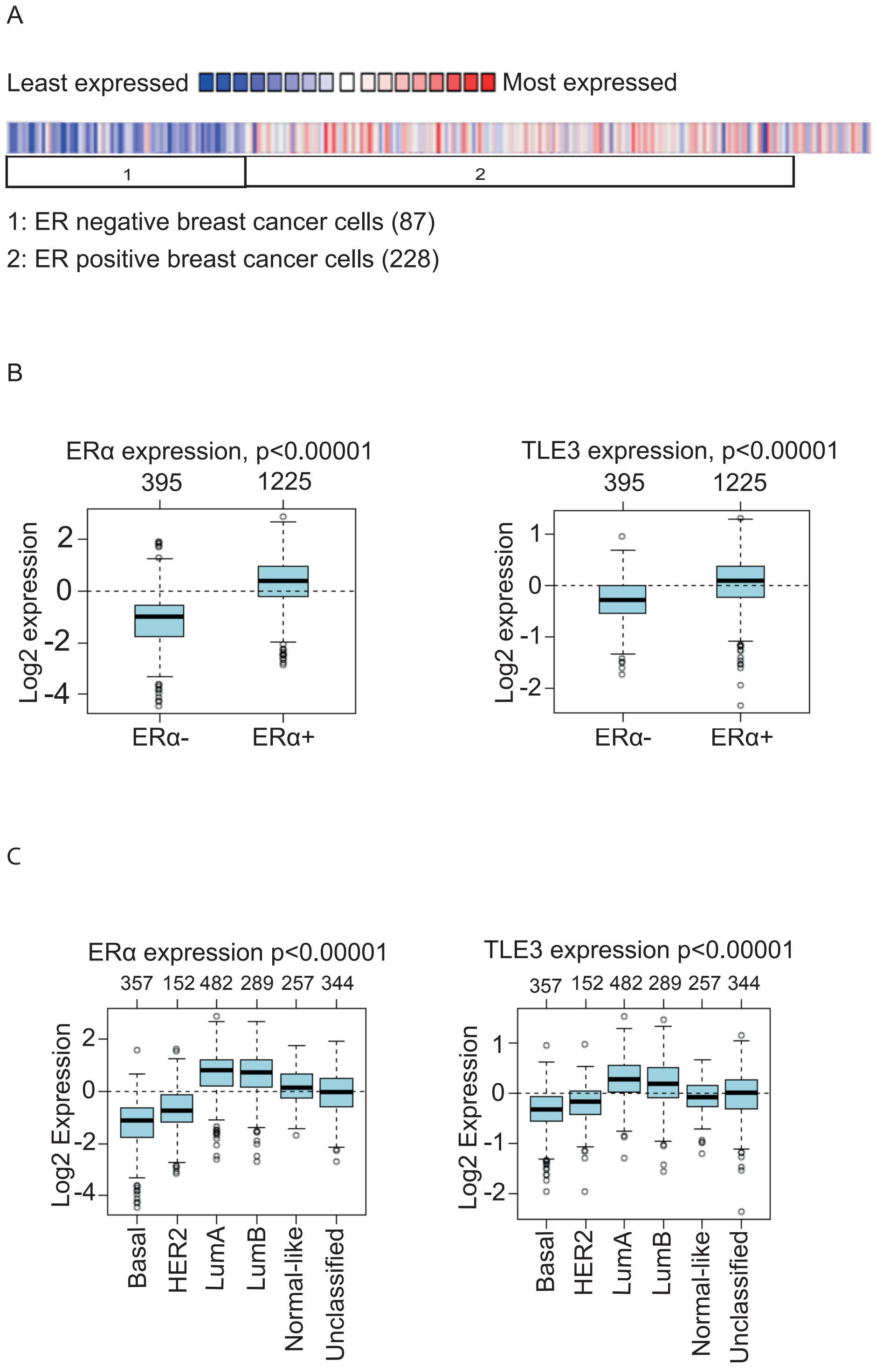

A

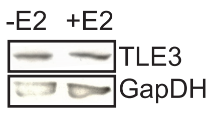

B

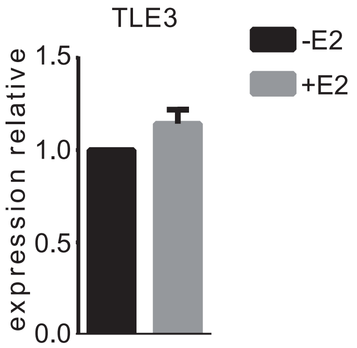

Jangal et al. 2014_FigS3.

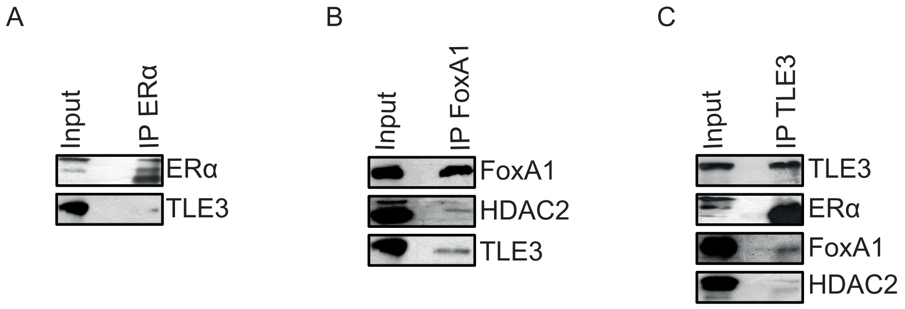

A

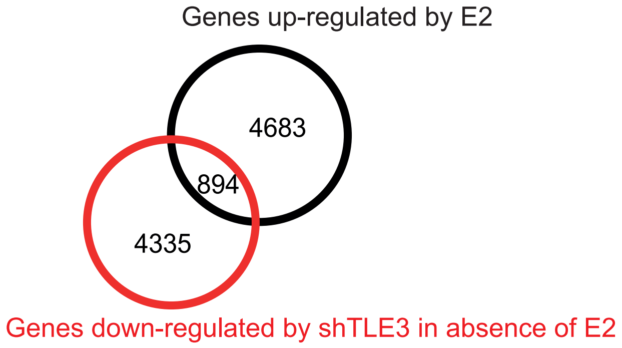
Genes up-regulated by E2
4683
894
4335
Genes down-regulated by shTLE3 in absence of E2

B

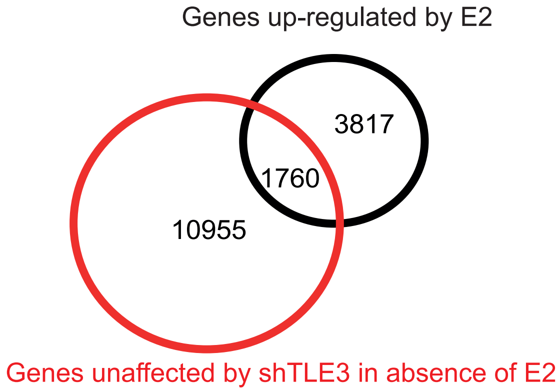
Genes up-regulated by E2
3817
1760
10955
Genes unaffected by shTLE3 in absence of E2

Jangal et al. 2014_FigS5.

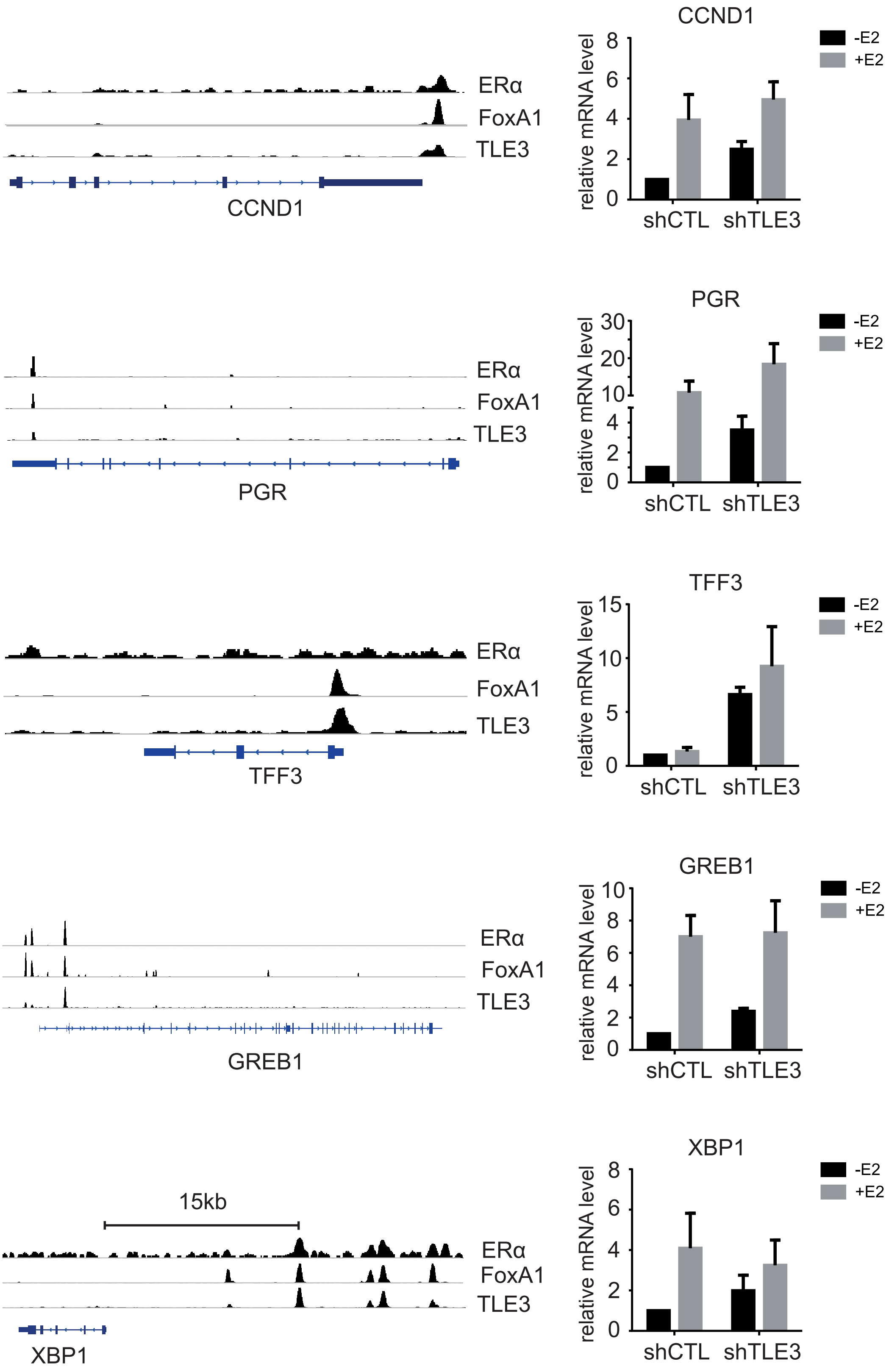

Jangal et al. 2014_FigS6.

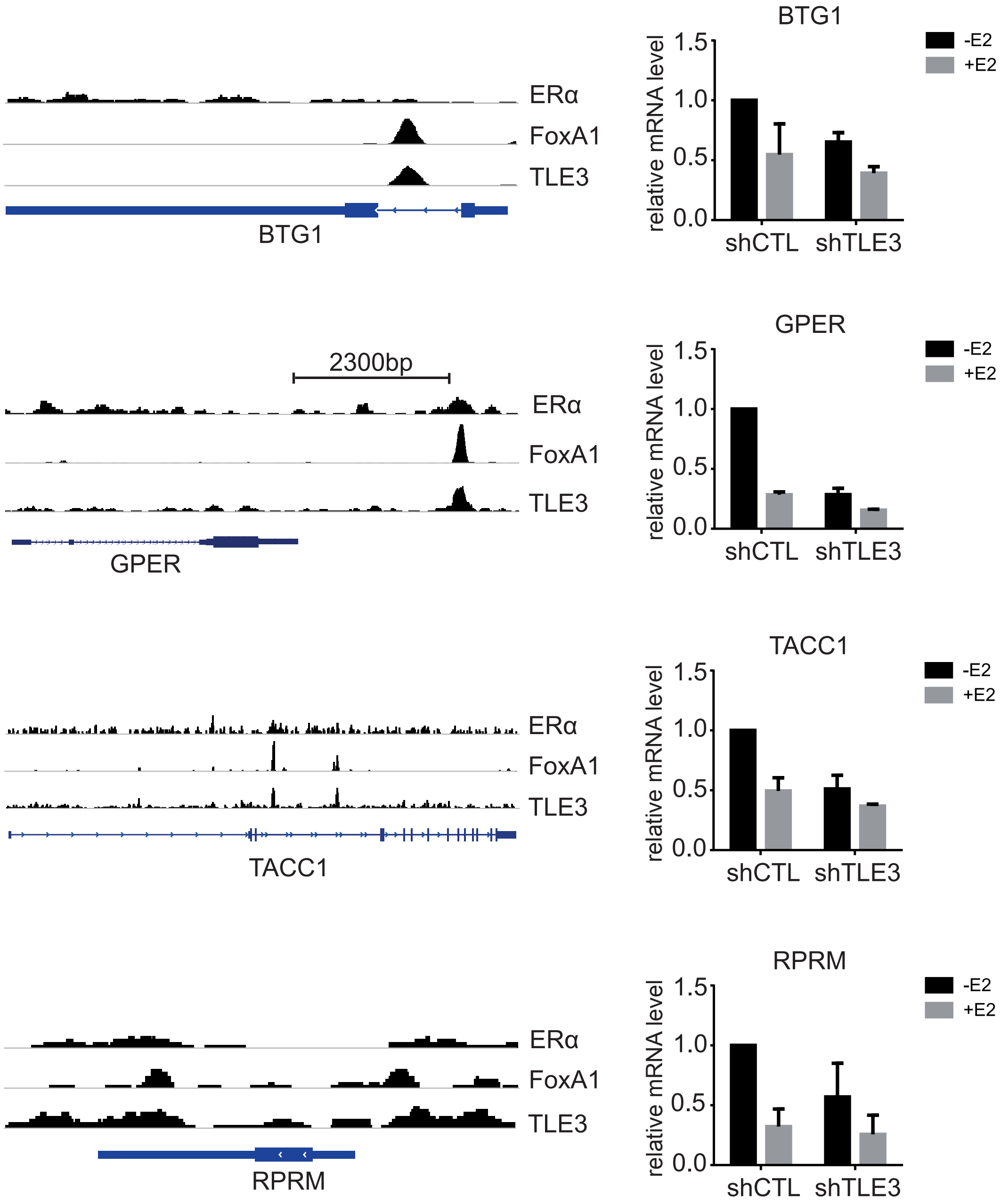

Jangal et al. 2014_FigS7.

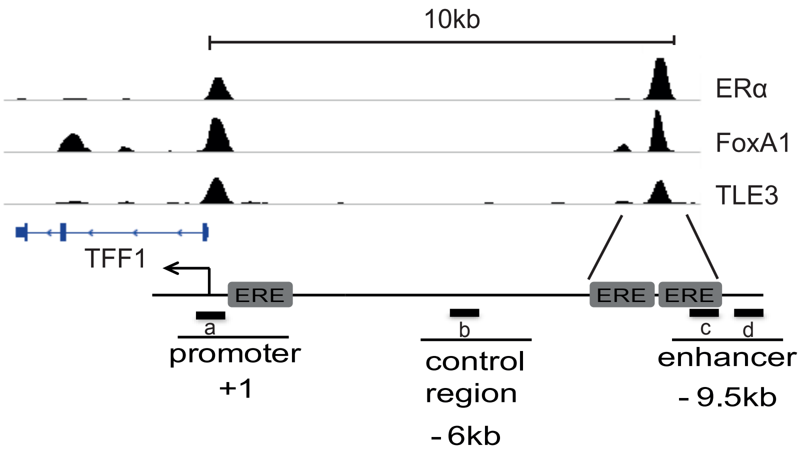

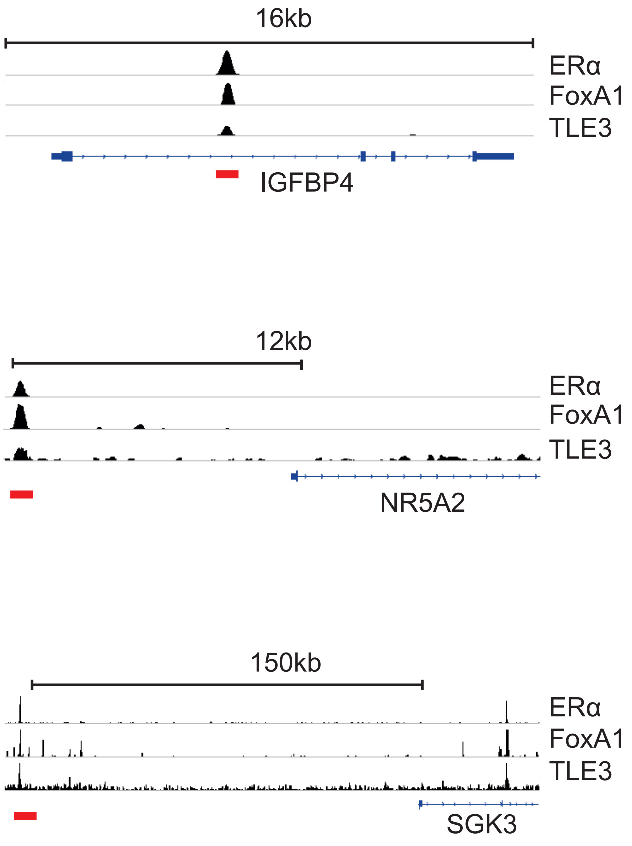
16kb
ERα
FoxA1
TLE3
IGFBP4
12kb
ERα
FoxA1
TLE3
NR5A2
150kb
ERα
FoxA1
TLE3
SGK3

Jangal et al. 2014_FigS9.

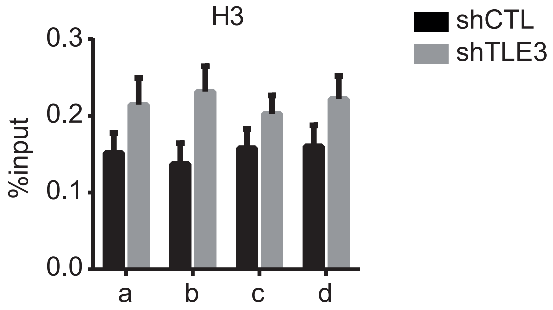

Jangal et al. 2014_FigS10.

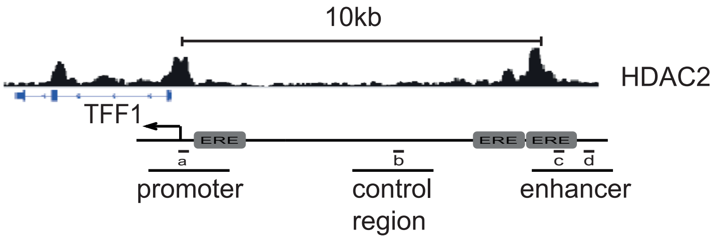

A

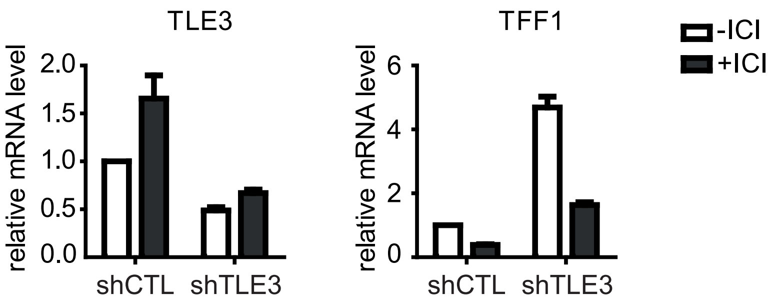
TLE3
TFF1
-ICI
+ICI
relative mRNA level
2.0
1.5
1.0
0.5
0.0
6
4
2
0
shCTL
shTLE3

B

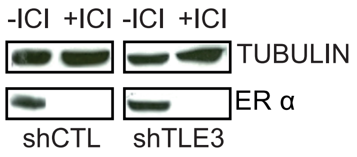
-ICI +ICI -ICI +ICI
TUBULIN
ER α
shCTL
shTLE3
